# Tendency of Misdirected Education and the Unbalanced Mind to Produce Insanity

**Published:** 1858-07-01

**Authors:** Edward Jarvis

**Affiliations:** Dorchester, Mass.


### ART. III.—TENDENCY OF MISDIRECTED EDUCATION AND THE UNBALANCED MIND TO PRODUCE INSANITY.

BY EDWARD JARVIS, M.D.,

*Dorchester, Mass.*

ALMOST from the beginning the risen generations have done what their intelligence, their means, and their conscience allowed them, to aid in the development and training of those who were to come after them, and to lead children and youth through their narrow paths to the highway of manhood. In the way that seemed to them best they have endeavoured to show what should be done with the untaught human mind, as it comes originally from the Creator,—the raw material of thought and intelligence, as it is delivered by Nature into the hands of its rightful possessor or his friends,—and how this should be wrought, shaped and furnished with knowledge of facts and principles, and fitted to bear the responsibilities of mature life. Many have given to their thoughts on this subject a visible form, and sent forth to the broad world treatises on Education, for the benefit of as many succeeding generations as will read them. These have all done, or are doing, their appropriate work, each in its due manner and degree. Generally they have one quality in common,—they treat of man as an integer, an identity composed of body and mind, and presuppose that all have similar powers and similar wants, and are to be educated in a similar manner. Most of them regard the intellect almost exclusively, and propose to fill it with knowledge of various kinds, which may be used for the various purposes of after-life. They propose by proper training to develop, and by suitable exercise to strengthen the mind, and give it power of concentration, energy to grapple with the subjects that may be presented to it, and a capacity to add to its stores of knowledge through its coming years. In this way the perceptive and the reasoning faculties, the memory and the imagination, are cultivated in various degrees, and gain thereby a varied measure of force. This is the usual extent of the plans of education. Even those which are called liberal, and are supposed to be expansive, are commonly limited to the development, cultivation, and discipline of these elements.

In as far as these plans of education are not founded on a proper and comprehensive view of the whole nature of man, and of the great and entire object for which he is placed in the hands of the educator, they fall short of their fulness of purpose; they overlook some of the parts or elements of the human constitution; they leave some of these undeveloped, some untrained, and others undisciplined. The teachers, wanting a thorough knowledge of the material on which they are to operate, and of the fabric which they are to create from it,—without a complete consideration of man in his natural and uneducated state, and of what he may and should be, of the dangers to which he is to be exposed, the burdens he must bear, the responsibilities he may be required to sustain, and the ends he may accomplish,—too often send their pupils forth to the world unfitted to sustain their part in its movements. And these youth, with a disproportionate development of their powers, and without a complete control of their own forces, with minds unbalanced, and wrong conceptions of their relation to society, err in their self-management; they fail to realize their own ideals of life, and are in danger of being overwhelmed with mental disorder.

*Comprehensive Plan of Education.*—A rational and a natural plan of education looks upon man not as a simple, but as a compound being,—not as a single integral power, but as composed of many and various powers. Among his elements are included not only the body and the mind, but the moral faculties and the appetites, the passions and the propensities. All of these together make up the man. Each has its own definite station to fill, and its special part to perform, in the human economy. In the perfect and healthy man these are all arranged in suitable proportions, and act in unvarying harmony. Each has its predominant, mediate, or subordinate place; each does its own work, and no more; and all co-operate for the good of the whole,—the health of the body and of the mind,—the elevation and happiness of the being to whom they belong.

In this perfect arrangement the moral power, the nobler element, stands above all the rest, and superintends the actions of the whole. The mental powers, like an intelligent overseer of a manufacturing process under the general charge of the proprietor, search out the ways, lay the plans, they direct all the organs and operations of the body, and control the appetites, passions, and propensities under the guidance of the conscience.

The powers that belong to the body are all necessary for the healthy operation of the whole corporeal frame, and for the sustenance and action of the mental and moral faculties here on the earth. Of these all are, in some degree, and a part of them are wholly, under the control of the mind, and to that extent they do its bidding. The appetites, the lower passions, and the propensities are active, or ready to be active, from the beginning. They crave indulgence, and, if left to themselves, they hardly know a bound to their gratification. But, being under the control of the higher powers, they are, or should be, restrained within their proper sphere. There seem to be several and various moral and mental powers and faculties, each of which has its special purpose to fulfil in the human economy, and all of which act in concert. Each performs its own appointed work, and no other and no more. Each has its due position and its due influence, governing, aiding, or obeying, according to the law prescribed to it. All of these attributes, or their germs, are given to man at birth, but not to all in the same proportion. Yet, with some exceptions, they are given to all in sufficient degree for the maintenance of health, and the fulfilment of the responsibilities of their present being. Some of the powers and attributes, as the appetite for food and drink, and the digestive function, are bestowed in full measure at the beginning of life. Of others only the primordial element is given, and these are subject to growth and development from infancy to maturity.

*Purpose of Education.*—It is the true purpose of education to draw out, cultivate, and strengthen the mental and the moral powers, and to subdue and discipline the appetites and passions. As in the healthy physical frame, the various organs of digestion, respiration, and locomotion,—the skin, brain, and nervous system,—are all in vigorous condition and action, none doing too much, and none coming short of its requirements, each receiving its part, but none demanding too much of the nervous influence, and each contributing its part to the sustenance and health of the whole; so, in the mental and moral constitution, the perceptive faculties, the reason, the memory, the imagination, the conscience, and all the lower powers, should each have its due development and influence, each its due energy and position, each be predominant or subordinate according to its office for the time being, and all act in concert for the good of the whole.

*Well-balanced Mind.*—This due development of each and all the mental and moral faculties, and their proportionate and harmonious action, constitute that which is called a well-balanced mind, such as belongs to one whose judgment is sound and reliable in all common affairs of life; who, from any given facts or propositions, is sure to come to just conclusions; who lays his plans of action in accordance with the measure and kind of his own strength, and with the circumstances amidst which he must operate; and who is certain, under any conditions, to do that which is right and appropriate. This well-balanced mind constitutes perfect mental health. It comes from original harmonious endowment, and proportionate development and discipline; that is, from appropriate education of all the powers. To this point it is desirable that all should arrive when they reach maturity, and are ready to enter upon responsible life, to take upon themselves their own self-management, and to perform their several parts in the affairs and duties of the world.

*Law of Growth of the Powers and Elements of Man.*—But the education of man is not finished, nor does the necessity of discipline cease with his youth. The growth of the bodily organs alone ends with that period. All the other powers—the mental and moral faculties, the passions, appetites, and propensities—have no such limit to their expansion. They may grow indefinitely even to the end of life, in old age. They may grow with accelerated and accelerating force, each step in the progress increasing the facility of taking another. Every one of these faculties and attributes of man increases in strength and activity by exercise, by use, by indulgence. The growth of the human powers by cultivation is a fixed law; yet it does not operate equally and in the same degree at all times, but with a constantly increasing force by successive repetitions. The longer the cultivation of any faculty or endowment is continued, and the more vigorously it is pursued, the easier its action becomes, and the greater is its accession of strength. The increase is added to the capital already existing, and the augmented capital allows still more rapid increase. As in the progress of fortune all the previous accumulations of money, property, or credit, become capital, by which more and more can be gained, so in the constitution of man all growth of any of the faculties, every new acquirement, every increase of force or discipline, every new elevation of purpose, is a new means of gathering more and more of the same kind; for the universal law of both nature and revelation, that "Whosoever hath, to him shall be given, and he shall have more abundance," operates in the intellectual and the moral constitution of man, as well as in his outward condition.

On the other hand, as in the decline of fortune every pecuniary loss, and every neglect to secure due and honourable advantage, increases the danger of another sacrifice, and diminishes the power of preventing it, so in the mental and moral constitution every neglect of study or discipline, every misapplication of intellectual force, every perversion of any of the faculties, every undue indulgence of any appetite or passion, every error or sin, increases the danger and the chance of the repetition of the same mistake or fault, and diminishes the securities against their influence; for "Whosoever hath not, from him shall be taken away even that he hath."

The practical operation of this law, both of growth and of decline, is manifested everywhere and among all men, and few are they who cannot trace it in some form or other, even in themselves, in the cultivation of any or all of the intellectual powers, in the study of language, mathematics, natural history, or any other branch of literature or science, in the cultivation of the moral and spiritual affections, the religious element, the conscience, the sense of right and wrong, regard to truth, love of man.

In the intellectual progress, the more one learns, the greater is his power of acquisition, and the taste for and the facility of acquiring increase with it. In the moral progress, the more the heart is warmed, the greater warmth does it demand to satisfy its desires; the more the spirit is elevated, the higher are its aspirations towards the true and the infinite. We see the same law in the cultivation of the tastes, the love of nature, of the beautiful, of music, of painting, of any of the fine arts. In these, indulgence creates strength, and strength gives enjoyment and a craving for more and more, and with these come the means and resolution to obtain greater gratification.

All the other faculties and powers, every appetite and passion, all the lower propensities, are subject to the same law of growth. Among the bodily appetites, the fondness for food, if gratified beyond the natural and healthy wants of nutrition, increases with indulgence; and this goes on, day by day, year by year, until the appetite may become the ruling element, and prevail over discretion and reason. The use of tobacco and opium is a still more marked illustration of the law of growth; for at first there is not only no desire for and no pleasure given by these, but even an absolute aversion to them. The mouth loathes, and the stomach is nauseated by them. Yet this aversion is overcome by persevering cultivation, and then a positive appetite for these narcotics arises, and this increases by fostering, until it becomes strong enough to govern those who use them, and to make them dissatisfied with everything else so long as this their ruling taste is not gratified. The desire for intoxicating drinks grows in the same manner, from small and apparently harmless beginnings, to great and even destructive power, when it subdues the whole man, body and soul, and compels the reason and the will to minister to its purposes. The sensual appetites, and all the lower propensities, obey the same law of growth, when indulged beyond the limit assigned to them by the reason and conscience. The passions, of whatever nature, the likings and the dislikes, the sympathies, antipathies, and caprices, all come under the same law, and, when left to follow their own course, uncontrolled by the higher element, they tend to expand and gain power beyond their healthy limit.

As the clay is in the hands of the potter to be moulded into such shapes as may please him, so the plastic elements of man are at first in the hands of his teacher, and afterwards in his own, to be formed and shaped as they may desire and direct. By cultivation of some of these elements, and by neglect and repression of others, one can make himself to be what he pleases. He may give his intellectual, his moral, or his animal nature a predominance. He may become a thinker, a reasoner, a sentimentalist. He may be a philanthropist or a misanthrope, an enthusiastic religionist or a cold-blooded atheist, a wise and sagacious statesman or a crafty politician. He may be a man of serene temper, generous, affectionate,—or he may be irritable, passionate, suspicious, hateful, selfish, miserly. He may be an eater, a drinker, a sensualist in any form, the slave of any appetite, the manifestation of any vice. He may be, almost entirely or principally, any one or number of them in various degrees, according to the way and extent his manifold powers and elements are educated by his teachers, by the influences that bear upon him, by his own self-management.

*This Balance of the Powers and Elements must be Maintained through Life.*—Each one of the powers, attributes, and endowments of man, being given to him for a definite purpose, each having a special station to fill and part to perform in the work of life, and the co-operation of each being necessary at all times for the proper and vigorous action of all the rest, it is requisite for mental health, and for the preservation of a well-balanced mind, not only that the appropriateness of position and a due proportion of all the intellectual and moral powers should be established during the process of development and growth in youth, but that they should be maintained during the whole of life. From the beginning to the end, each faculty and power should be cultivated or chastened in its due degree. None should be allowed to become excessively strong and active, while others are weak and dormant; none should absorb the force that rightfully belongs to the rest. The higher elements, then, should always be sustained in their commanding position, and the lower should be kept subordinate. The appetites should be indulged, and the propensities allowed to act, only at such times, and in such periods, and so far, as the health of the system requires; and all the passions and the moral affections should be applied to their legitimate purposes, and to no other. All should be measured, directed, and controlled by the reason, which should reign paramount over these, and yet, in its turn, be the faithful servant of the conscience, rendering it a never-failing and implicit obedience.

This condition of mental and physical health requires—1. Great discretion to determine what the proper arrangement of the faculties or elements of power is, and what their several forces should be, in order that they may make up the perfect man; 2. Constant self-analysis through life, to see whether this due order and proportionate power is maintained; 3. An unfaltering self-supervision and self-discipline, to maintain, in their proper position and relation, all the elements of our constitution and frame, encouraging the higher, directing the mediate, and chastening the lower.

*Balance of the Powers Disturbed in some.*—In manifold ways men fall short of this perfect standard of mental condition. In some, the deficiency is so slight as to produce no apparent effect on their soundness of mind; in others, it is so great as to produce manifest insanity; and between these two extremes there are all intermediate grades of unsoundness. The slighter variations from this normal standard are very frequent. Even after one has been properly educated, and enters upon maturity, there may be, and there commonly is, some one or more of the powers developed and strengthened beyond the rest, in connexion with some special employment, in the pursuit of some study, in the cultivation of some salutary taste for good, or in the indulgence of some passion or appetite for evil. Thus, in one man, the perceptive faculties are prominent and most active; and he has a quicker eye or ear, and more readily understands what is presented to him, than the average of men. In another, causality or the reasoning faculty prevails, and he quickly sees the relations of things. He traces events back to their causes, and follows causes onward to their results. In a third, conscientiousness predominates, and he is scrupulously fearful of doing wrong. In a fourth, benevolence is the favoured faculty, and he sympathises with suffering more keenly and readily than others. In another, wit is cultivated and made more active than the other powers, and he has a quick perception of the ludicrous, and of singular and droll analogies and relations.

All these, and all the other powers or modifications or combinations of powers, may and do receive in different persons extraordinary cultivation, development, and strengthening, in addition to the original and appropriate education of the whole. Thus men qualify themselves for, and become expert or skilful in, the various professions and arts of life, without diminishing their good sense in the common affairs of the world, or impairing their balance of mind. Nevertheless, although these minds act well on ordinary subjects, yet they act better on those to which they are frequently directed, and on which they are habitually employed. The mind always runs more readily and easily in its most accustomed channel.

We not only labour more easily and effectually on those subjects and in those ways which habit has made familiar to us, but there is a degree, and in some a great degree, of danger that the tone or character of the thoughts applied to these will tinge or modify those which we apply to other subjects. It may control the associations of ideas, and give its peculiar colouring and estimate to all others.

The *imagination* is naturally among the most active elements of the mental constitution. It tends to influence the associative faculty, and govern the inlets of ideas. It is the foundation of a great variety of mental error, and often at variance with discipline. It is therefore a very unsafe guide to life and principles. It needs the constant aid of the perceptive faculty to correct it, and of the reason to control it. The *law of association* is a manifestation of its power; circumstances, things, and ideas are suggested according to their natural or artificial connexions. The habit of associating them together gives them an affinity, so that they rise up in the mind in the same series of thoughts. When one is presented, the others follow; and the whole of a familiar scene, or train of circumstances, or range of ideas, follows the presentation of one of their elements or parts. Thus we are reminded of tales, events or trains of facts, by the mention of some single incident similar to any one connected with those that are thus suggested. In such cases, the memory and the associative faculties, which are required to move or act only in an old and familiar course, are more active and energetic than the perceptive faculties, which are acting or endeavouring to act upon a new subject.

While, therefore, the perceptive faculties are trying to present to the mind certain new images, the associative faculties present some old images, and these, mingled together, form a compound idea, consisting in part of the object last presented, and in part—perhaps in great part—of old and remembered objects, which are sufficiently similar to the new to be suggested by it. In these cases the perceptive faculties recognise and convey to the mind so much of the new image as is similar to old and familiar images; but at that point their action ceases, and the mind receives no more ideas through them, but the memory and the imagination fill up the rest of the picture.

From this cause we readily discover resemblances in things which we see for the first time, or with which we are but little acquainted, to those with which we are familiar. Thus, when one goes from his father's house, and dwells among strangers, he meets many persons who look to him like others whom he has left behind, and he is continually reminded of his home by their similarity. But, after he becomes familiarly acquainted with the new people and circumstances, he fails to see the resemblance, and wonders how he could have seen it before.

This is easily explained by the law of suggestion and the activity of the associative faculties, the memory and imagination, which is greater than that of the perceptive faculties. The home-sick boy's mind is filled with the objects that he left behind; their images are familiar and dear to him, and the slightest prompting calls them up. Meeting a stranger, he sees some feature, expression, or manner,—like a feature, expression, or manner in some one at home. All the features, person, and manners of the absent are associated with this single feature which is thus presented, and are suggested to him by it. Here the perceptive faculties stop, and the imagination fills up the rest of the picture — not with the other features of the person before him, but with those which are familiar to his mind, and dear to his heart.

But after he becomes acquainted with persons of the new place, and his heart is reconciled to those who are about him, and weaned in some degree from those with whom he lived before, the perceptive faculties become more, and the associative and suggestive faculties become less, efficient. Then, when he meets these persons, he sees more and more of their real features, and thinks less and less of those who seemed to resemble them. The outline is filled with the things before him; and that point which alone he first noticed, now bears so small a proportion to those which he now sees, that he finds none of that resemblance which he saw so readily before.

*The Ruling Feeling or Interest Colours New Ideas.*—According to the same law, any ruling feeling or interest directs or controls the perceptive faculties in greater or less degree, and infuses itself into, and modifies, the images that are received from any sources. The same object, presented to several men who have different predominant feelings or interests, will suggest as many and as various images. In the same landscape, the arrangements of the fields, the gracefulness of outline and detail, present to the painter a fit subject for a picture. Its soil suggests to the farmer the idea of its fitness for cultivation of various crops; the speculator sees its appropriateness for building lots; the geologist, the composition of the earth; the botanist, the various kinds of plants that grow upon it.

In all these and similar cases, the ruling idea, whatever it may be, directs the perceptive faculties in some degree, and compels the eye to see, and the ear to hear, and the mind to perceive, that which is in accordance with itself, and prevents them from recognising that which is not in harmony with it. More than this—it accepts the suggestions of the memory and the imagination in place of the present realities which the perceptive faculties, uncontrolled by such influence, might have discovered.

For this reason, witnesses, who testify for opposing parties and interests in courts, may very honestly give very different accounts of the same occurrences or things which they both had seen. Each one saw and perceived the most readily that which was most consonant with the previous feeling or interest; and these modified the remaining perceptions, and controlled the inferences.

Even philosophers, or those who intend to be philosophers, are sometimes subject to this error in their investigations. If they adopt a theory on any subject, its influence, to a greater or less extent, controls their perceptive or reasoning faculties. The former most readily, and perhaps exclusively, recognise those facts which are in harmony with the preconceived idea; the latter draw conclusions corresponding to it; and the imagination fills up all the vacancies in the picture. Hence, these men are apt to find confirmation of their doctrine in their discoveries. And even men having opposite theories of the same subject are in some danger of confirming each his own from the examination of the same facts.

The moral affections and the passions have a more powerful influence in controlling the perceptive faculties and the reasoning, than even the pre-occupation of ideas. We delight to clothe those whom we love with the raiment of beauty. We see in them virtues and powers which less partial friends cannot discover. The evil passions have more absorbing power, and a more complete government of the channels of ideas. When one is excited with anger, or when he permanently hates, the eye is slow, and even blind, to discover virtue, propriety, or reasonableness in the object of his ill-will. Seeing through the preconceived idea, he clothes this object with evil and wrong; then reason is suspended, or made to subserve the passions, and to aid in establishing conclusions corresponding with his predominant emotions, and these compel him to utter language he would not have spoken, and to perform deeds he would not have found a motive for doing, when not under the influence of passion.

*Effect of Habit on Mental Action.*—Whatever power or element is accustomed to action, acts more easily than such as have lain comparatively dormant; and in whatever way any of the mental or moral powers are used most, they find more ready action there than otherwise. This is the most agreeable, as well as most easy, and our feelings prompt us unconsciously to let our thoughts run in this course.

These imaginative habits sometimes become very powerful, and require vigilance and self-discipline to control them, and prevent their controlling us. The mind of a student, who has great facility in making puns, runs so readily and insensibly in this way, that sometimes, when he attempts to study, he finds it difficult to prevent his analysing words, and forming new combinations of syllables, to make out some new and strange meaning.

*Unbalanced Mind.*—Although all of these are consistent with what is usually called mental health, yet such men have a disproportionate distribution of mental force; some ruling idea has undue prominence in, and often undue control over, the mind, and they are, in certain ways, unbalanced; still, as they retain their reason, and can correct their error of judgment by comparing their false perceptions and conclusions with those which they know to be true, they are presumed to be sound in mind.

*Danger of its Growth.*—As all habits and powers, all passions and propensities, are liable to grow by exercise, every one of these irregularities may, by cultivation or indulgence, become so strong as to overcome the reason, and cut off the means of correcting mistakes in judgment, and thereby establish insanity. It is the first step that costs; the others are most easily taken. The only absolute security for the mental balance is in the utter avoidance of even the least perversion of thought or feeling.

Some are led to begin this course of error by distinct and well-marked tastes for it. In others, a feeling is accidentally excited; it may be very slight at first, but by repetition it gains strength, and ultimately becomes powerful. This is remarkably manifested in the caprices and perversities. The mind capriciously determines to be pleased with a small point, and through this sees all the rest. This prepossession compels the perceptive faculties to present the acceptable trait first to the mind, and put it in good-humour to see those associated with it, and then it looks upon them at least with toleration. By repetition, the toleration becomes satisfaction, and approbation follows after. At last, the whole mind is brought under the power of the caprice; then opinions are formed, and a course of conduct pursued, from which the reason at first would have shrunk; but being disarmed and made the servant of passion or caprice, it goes to strengthen the error and overthrow the judgment.

*Day-Dreaming.*—The day-dreamer loves to form an ideal image of that which he would like to be, and of that which he would wish others be, or of what he would like to have done. For this purpose, the images derived through his perceptive faculties are only used as suggestions of better images, or better arrangements of facts and circumstances; something unreal indeed, but more satisfactory than that which is presented to his senses. In this the reason is suspended, for there is no wish to make the ideal image correspond with any rule of truth. Comparison is set aside, for no known standard is to be the measure. But the dreamer is at liberty to create whatever he will, and this he does in a form and manner most agreeable to his taste and his ruling element. Thus he improves upon the circumstances, or acts, or speeches that are presented to him, and frequently makes himself the principal actor or speaker in the scene of his new creation.

This habit belongs to those who have large self-esteem, or large love of approbation, more than to others; they love to form desirable scenes of distinction, of influence, or even of glory, in which they place themselves. From the little boy who delights to imagine himself the drummer of the train-band, up to the man who indulges the dream of his being a commander, an orator, or philosopher, there are all stages of progress, and all grades of imaginary life and position.

At first, and in some, this may be an honest conception of improvement upon that which is seen and heard. When one sees some work performed, he may readily imagine a better way, and think that he would do it according to the ideal. If he hears a speech, he may conceive of a better argument and an improved series of ideas, and he would so present them if he were the speaker. It is a reasonable gratification to conceive of images of perfect virtue or noble action. One, therefore, easily allows himself to create this ideal of life and thought, and even to place himself in the centre. It is so pleasant to see one's self in a satisfactory position, that the dream is again indulged. By repetition it becomes more and more easy, and even attractive, and then those who have fallen into the habit find it difficult to escape from it. It is hard to fix their attention exclusively upon the realities of life, and prevent their thoughts from wandering to imaginary scenes, where all is satisfactory, but where none is actual, and but little is true.

*Knowledge to be Acquired in Youth.*—Beside the work of development and discipline, of harmonizing the several elements of the mental and moral constitution, of establishing each in its due position, and giving to each its proportionate and appropriate force, it is the further purpose of education to instruct the youth in facts and principles, to teach them their own nature, their relation to the world and to outward things, and their responsibilities in their several positions, and to fit them to discharge the duties that must come upon them. It should also prepare them to exercise a constant self-control, and to apply their powers, on all occasions, to proper and desirable purposes.

*Defective Plan of Education.*—Notwithstanding this plan of education seems not only reasonable, but absolutely necessary for the fulfilment of its object, yet many come short of it, and include only a part of these requisites; and others are still more meagre, and include within their scope none of those things which are the support and direction of every man and every woman, in their true and successful walk through the earth. However valuable the knowledge they impart may be, still the one thing needful—the knowledge of themselves and of life, of external nature and of man, and the relations of these to each other—is not given; and the pupils who are thus trained are sent forth to grope their way through the world, without that light to guide them, and to struggle under their responsibilities of life, without that strengthening and discipline which they should have received at school in their early years, and the forming period of their existence.

In these systems of education, it is interesting but painful to see how many needless things are carefully provided, and faithfully done, and how many necessary things are entirely omitted; and when the teachers have finished their work, and the pupils have acquired all that is offered, it is mortifying to see how little it can avail them in bearing the burdens and discharging the duties of life. In these schools the scholar may accumulate the vast treasures of knowledge, and yet be poor indeed in all that will establish and sustain him in the position of life, health, success, and happiness, for which he seems to be destined. He may fathom the depths of chemistry, and analyse all compound substances of earth, of vegetation, of animals, and spread before his clear vision their secret elements. He may know of what the mineral, the plant, and even flesh and blood are composed, and yet be ignorant of the elements of his own constitution—of the nature, extent, uses, limits, and liabilities of his own powers of body and mind, of emotion, and of passion. He may comprehend all the principles of material philosophy, and the measure and character of the natural powers, and understand how to bend them to his purposes. He may master the elements, and compel the waters, the air, steam, gases, electricity, to lend their forces and labour at his will: he may make them bear his ships, turn his machines, and carry his messages, and yet know not the nature and use of his own vital machinery, nor how to apply his own internal forces, to control his appetites, and govern his passions. All external nature may be made to serve him, and do the work of his bidding, and he is successful in his plans connected with it; but the elements of his own being, body and spirit, are not at his command, and in his endeavour to use them, and gain and enjoy health, and sanity, and duration of life, he fails, because for these he was not prepared.

There are other plans, or rather customs of education, far worse than these. Their sins are not merely those of omission. They teach not merely facts that are useless, and principles that have no practicable use, but they teach positive error. They give wrong notions of life. They excite expectations which cannot be realized, and lead their pupils to form schemes inconsistent with the circumstances which must surround them. One of the common faults of such education is to develop and cultivate unfounded hope and ambition, rather than discipline and laborious patience. Under this system, youth are induced to form purposes which they have neither the strength nor the industry to accomplish, and for which they have made and are making no suitable preparation. They are encouraged to look for a degree of success in life, a measure of prosperity, of respect, and of influence, which they have neither the talent, nor the wisdom, nor the power of adaptation to obtain. Their expectations are rather in accordance with their desires, and perhaps their self-esteem, than with the fitness of their plans, or their perseverance in accomplishment.

Starting with wrong notions of life and of their own relations to the world, and with false conceptions of things as they are, they err in their purposes and expectations of present existence, and in their ideas of self-management, and fail to adapt their plans of action to the opinions and customs of other men, and to the circumstances of the world amidst which they live. Deficient in that good common sense which would always establish and maintain a true and certain relation between their own ideals and the realities of the world, they frequently fail in one unsuited purpose, only to enter upon another alike unsuited. Of course disappointment follows them, because they expect impossible results, or neglect to use the due means and energy to obtain them. Experience does not teach them wisdom, and they do not learn, from one failure, how they may avoid another. Successive defeats distress and confound them more and more; they become less and less able to adapt themselves to things as they are; until, at length, some of them sink into hopeless confusion, and others into mental disorder.

*Want of Plan of Life.*—There are some who have no settled plans of life to follow—no determined purpose to fulfil. They are deficient in firmness, and unwilling or unable to persevere in what they undertake. They enter upon schemes without a clear conception of what their ends should be, or how they should be accomplished. They are often weary of their purpose, and leave it even when it may be approaching a successful issue. Wanting a balance-wheel in their mental machinery, they are governed at one time by one motive, and at another by a different one; or, undecided which of two or more diverse motives to obey, they follow one in part, and another in part, but yield fully to and derive advantage from neither. In their indecision, they sometimes adopt several contradictory or irreconcileable plans, and of course they fail in all. Thus they are turning from purpose to purpose, floundering amidst difficulties and unyielding circumstances, striving in vain to make opposing plans and conditions harmonize together.

*Indiscretion.*—Akin to the last class are the indiscreet, who likewise labour under a disproportion of mental development and action. They have indistinct perceptions, but are impatient of investigation. They have active imaginations, which to them seem to compensate for the want of persevering cultivation of the perceptive faculties and of cautious comparison. They have a habit of rapid deduction, and draw ready and bold inferences from few and insufficient data. They are the people whom the philosopher describes as learning a few facts, guessing at many more, and jumping at a conclusion. They form their opinions without knowing or considering all, and perhaps not even the most important, facts that should be regarded. They arrange their plans and conduct their business, they manage themselves and their affairs, with the same imperfect regard to the facts and circumstances that should govern them, as they manifest in the formation of their opinions, and they are necessarily unsuccessful.

On account of their loose habits of reasoning, and proneness to form hasty opinions, these are considered by their associates as men of unreliable and even unsound judgment. Their mental condition is not insanity, but, in some of its phases, there is a great similarity between them. There is a want of a due distribution of force and activity among their mental faculties. They especially lack the necessary activity of the reason to correct their errors of judgment. And though their opinions may be often changed, they discover no mistake in the process through which they are formed. This class, therefore, rarely improve. On the contrary, there is danger that this disproportionate activity of their imagination and slowness of their reason will increase, disturbing the balance of their minds more and more, and rendering their judgment less and less sound through the progress of years.

*Love of Excitement.*—The unbalanced mind is sometimes manifested in love of excitement—in the uneasy restlessness of those who do not find sufficient motive of action in the ordinary affairs of life, and the usual interests and affections of home. These persons crave something out of the common course. As the intemperate want alcoholic drink to stimulate their bodies to action, and feel languid without it, so these desire some enlivening circumstance, event, or company, to give activity to their minds and buoyancy to their feelings.

At their homes, and in their own families, they are comparatively languid and listless. Some of them are not interested in domestic affairs; and, when no strangers are with them, some are careless as to their manners, and negligent as to their dress. Interested in no occupation, they dawdle away their time, which, for the want of satisfactory employment, passes wearily onward from one opportunity of indulging their excitability to another. When in company or abroad, they are lively, bright, and joyous. Their spirits are full of energy, and their minds are active, and they are acceptable companions in society. But when they return to their homes, or when their company departs, they sink again to their usual languor and indifference. Many of these are fond of amusements, and especially those of a public nature. They love the theatre or concerts; they frequent the lecture-rooms, or other places of general gatherings of the people; they are found in places of public promenade; they take advantage of whatever opportunity may be within their reach to indulge their taste for new means of excitement.

Some demand even greater changes than these: they want changes of home. At one season, they go on distant journeys; at another, their dwelling is at the sea-shore; and anon they visit the mountains. They go from the city to the country, and from the country to the city. These changes, which the well-balanced mind only wants as occasional relaxations from protracted labour or care, seem to the restless lover of excitement to be necessary aliment of satisfactory life. Others are more quiet in their physical habits, but yet have the same mental restlessness. Some find means of gratifying their excitability in reading novels and tales of thrilling interest, some in reading newspapers, some in the agitations of politics, in hearing and telling news, in the gossipry of the neighbourhood.

This varying course and habit of life, the alternations from excitement to languor and from languor to excitement, successively, is exhausting to both the physical and mental constitution. If the excitability is indulged and cultivated, it grows more and more; the mind becomes more dependent on some external and stimulating influence for its lively enjoyment, and grows more languid in the interval, and then the ordinary affairs, the humdrum of every-day life, grow less and less interesting, and even burdensome; the mind is dull, and the temper may become irritable and peevish.

After years of this indulgence, in some persons, pleasures, company, and novelties pall upon the heart; the mind is wearied with that on which it feasted before, and sinks into permanent languor, or becomes so unstable in action that reason loses its power by any effort to direct it.

*The frivolous* have similar elements of error. They have no elevation of purpose, no stability of character, nor perseverance in action. They are satisfied with small and temporary matters. They are unwilling to take upon themselves the heavy responsibilities of life and society. They trifle with serious things, and treat grave interests with levity. Their delight is in present amusement, the idle occupation of the hour, and beyond this they feel no anxiety. Their unbalanced minds wither with their exhaustive activity, and they faint beneath any burdens that may be laid upon them. The pursuit of pleasure and all amusement, when followed as a principal object, and not as an occasional relaxation from the business of life, both tend to the same result —they waste the mental powers, and exhaust the moral force, and leave their devotee in a state of helpless imbecility.

*Eccentricity.*—A fondness for notoriety is a tempting passion for some, but it is dangerous to the balance of mind, and often destructive to mental soundness. A perverted taste, a false estimate of themselves and of mankind, or a desire in some way or other to be noticed, leads some to assume habits of thought, or speech, or of body, which will distinguish them as different from the world amidst which they live. From the man who burned the temple of Ephesus that the world might know and remember him, to the College youth who kept a coffin in his room to make his acquaintances stare, men have sought, in manifold ways, to attract attention, and to impress themselves upon others. One is habitually gruff in his manners; one violates the ordinary forms of politeness. Another is peculiar in the form, or colour, or material of his clothing. One affects to be remarkably sincere, and gives opinions and states facts out of place and out of season; or he loves to differ in opinions on ordinary matters, and to say strange and startling things, or, by some other singularity of thought, or language, or conduct, he manifests his eccentricity to the little or great world who surround him.

The greater part of these peculiarities are voluntary, at least in the beginning, but they are established by repetition; habit makes the eccentric mode of speaking, or thinking, or action, the easiest, and then, perhaps, without intention, or even thought, the odd man presents himself in this manner to his associates, with little power to control and direct his thoughts and actions as other men do. In this class there is a want of mental discipline, a defective action of the reasoning faculty. They do not compare themselves with others; or, if they do, they do not see that, although they attract observation, they fail to secure respect and confidence. They do not discover that the world values its own opinions and customs most, and that whosoever violates the least of the requirements of the average common sense makes himself suspected of a liability, at least, to violate any or all of even the greater matters of that law, and is to that extent unsound in mind.

There is a natural and a just ground for distrusting the soundness of the judgment of those who allow any sort of oddity in themselves, or in whom it is even involuntarily manifested. If the reasoning faculty is resisted and set aside in one thing, it may be in another. If self-esteem, will, or caprice, rise above it at any time, and claim to interfere with the balance-wheel, they will do the same at any other time, whenever occasion may seem to them to require. The reason which is dethroned, or the judgment which is impaired, in connexion with any eccentricity that is adopted or allowed, loses the certainty of its paramount authority, and may fall again at any time.

*Self-esteem.*—Self-esteem in many ways disturbs the mental balance. It makes self the most active principle of faith and action. It gives a value to whatever proceeds from, or is connected with, self. It makes the perceptive faculties and the reason alike its servants. It allows the one to discover so much as is in harmony with it, and the other to make only such comparisons as will exhibit self to the best advantage, and never that which would mortify it.

Believing in themselves first, those in whom self-esteem is active are averse to laborious investigation and the slow process of reason, for they feel that they are sure to be right in their conclusions, whatever may be their foundation. They therefore draw inferences boldly from new facts, and form opinions freely upon subjects of which they have but little knowledge, and adhere to them with firmness, and speak of them with confidence. They are opinionated, and love to talk oracularly. They are sometimes fond of argumentation, and desire to impress their opinions upon others; and thus they become dogmatists. But their careless habits of reasoning and induction fail to convince others of that in which they have undoubting confidence.

They are impatient of contradiction, because that is an impeachment of their fundamental principle—faith in themselves. They are apt to become boasters, for they think their own acts and acquirements are as important to others as, in their own eyes, they seem to be. Striving thus to grapple with subjects which they cannot understand, or which they do not use the proper means to master, struggling in positions where they must often fail, their minds sometimes stagger, their mental balance may be entirely lost, and need a healing process to restore it.

*Malignant Passions.*—All the evil passions—anger, violent temper, hatred, malice, envy, and jealousy—are even more injurious to the balance of the mind than any of the merely mental disproportions. While these are in action, they absorb the whole man, his emotions and mind. They direct the perceptive faculties, they control the reason, and subvert the judgment. A man in a passion sees in the object of his anger those qualities, and only those, which he wants to see, and his imagination fills up the rest with such as correspond to his own state of feeling. He clothes his antagonist in a garb of his own creation, and then finds undoubted proof that he is wrong. The one offensive point stands for the whole, and those which are true and acceptable are overlooked. The paroxysm of rage may be but momentary, yet it is violent, and gives a shock to the whole mental and moral constitution. The feelings remain disturbed, the reason does not at once regain its ascendancy, but continues, for some time, the servant of the exciting and the maddening passions.

Malignity, hatred, jealousy, and envy are less violent, but more abiding. They have the perceptions and the reason less exclusively under their control, yet they have these powers more or less at their command, and influence the judgment. They enter into, and form a part of, the estimate of objects. They certainly disturb the balance-wheel of the mind, and leave it to run irregularly and uncertainly.

Let us now hear the conclusion of the whole matter. All the original and natural endowments of humanity, the mental and the moral powers, are distributed unequally among men. These are frequently irregularly developed, disproportionately exercised, and are often misapplied; they therefore need great discretion for their education in the beginning, and constant watchfulness and discipline for their government through life. The lower powers—the appetites, the passions, and the propensities—are by nature sufficiently active, and constantly seeking gratification. If indulged, they grow to an unhealthy extent. In some they grow exorbitantly, and even destructively. Therefore, they constantly need the control of reason and the supervision of the conscience to restrain them within the bounds appointed to them for the good of the whole.

From all these causes, singly or combined in many complications, there arise manifold varieties of waywardness, which we meet, in some form or other, in every society.

In all these persons the balance of mind is more or less disturbed, and the soundness of judgment is more or less vitiated.

From all proceed at times, opinions, language, or acts, that, taken by themselves, would be deemed insane.

All these perversities are subject to the law of growth by indulgence and cultivation, all disturb or weaken the reason in various degrees, and all tend to overthrow it completely and produce an acknowledged insanity. The danger of those who allow them is not outward, but inward. Their enemies are they of their own household. They go from strength to strength of waywardness, and from weakness to weakness of judgment, until it is lost.

The whole of these classes which we have here described constitute a pyramid of error. The lower stratum, or larger class, is composed of those who are educated imperfectly, or for undue purposes of present being; in whom some of the mental or moral elements are left dormant, and others energized and quickened to a disproportionate action; whose education either negatively fails to fit them, or positively unfits them, for the world and its unavoidable circumstances. The next stratum is composed of those who start with, or at any time adopt, wrong notions of life and of its responsibilities—of what they may gain, and of what they must endure.

After and above these are those whose minds, in the progress of life, from manifold causes, and in numberless ways, become unbalanced to a greater or less extent; who are struggling to accomplish impossible purposes, or to gain things beyond their reach; of whom some are quailing in disappointment or withering into weakness, and others are approaching, or even standing upon, the confines of mental disorder.

The apex of the pyramid is crowned with those whose reason has fallen in the struggle, and in whom insanity is established.

Considering how richly nature has endowed humanity, and how long, perfect, and happy a life she has offered to man and to woman the means and the opportunity of obtaining, by education, by instruction, and by self-discipline, it is melancholy to see how many there are who belong to this pyramid of error, of weakness, and of perversity. There are few persons of so limited observation as not to find within their own range some who are walking in these dangerous paths of waywardness—whose minds are in some measure unbalanced—who are in some degree the subjects of passion, and temper, and propensity—who are more or less influenced or even governed by caprice, undisciplined feeling, or unfitting desires.

Some of these have little or no firmness of purpose—some are immovably obstinate, wilful, and headstrong — some have no plans of life, and others have plans that cannot be reconciled to the circumstances that must surround them. In some there is a restless seeking for that which they cannot obtain, or which they cannot enjoy when they reach it. Some give an undue importance to whatever interests their feelings, and make mountains of molehills; others frivolously trifle with grave matters, and make molehills of mountains. But they are all travelling in that road everywhere strewed with error and failure, and where insanity often lies; and although, perhaps, only a small portion of them may arrive at that terrible end of reason's reign, yet they are all, in greater or less degree, unsound in mind; they are all, more or less, prominent candidates for lunacy; and no one is safe who thus allows his mental balance-wheel ever to be disturbed.

The general attention is so little directed to these dangers—so few are educated to meet and escape them—the public conscience is so little trained to feel responsible for mental health, that when insanity, through any of these ways, comes upon one, the friends are taken by surprise; they speak of the mysterious ways of Providence, and wonder that one so gay, so hopeful, should be bereft of reason.

But, as the abundant weeds and the stinted grain in the farmer's field are plainly chargeable to negligent or unskilful cultivation, or as spendthrift habits lead to poverty, so the insanity of many is plainly referable to the misdirected education which their parents gave them, to the unfitting habits which they established, or to the unbalanced mind which they cultivated.

---

